# Knowledge and attitudes toward COVID-19 vaccination in Sudan: A cross-sectional study

**DOI:** 10.3934/publichealth.2023023

**Published:** 2023-05-06

**Authors:** Safaa Badi, Loai Abdelgadir Babiker, Abdullah Yasseen Aldow, Almigdad Badr Aldeen Abas, Mazen Abdelhafiez Eisa, Mohamed Nour Abu-Ali, Wagass Abdelrhman Abdella, Mohamed Elsir Marzouq, Musaab Ahmed, Abubakr Ali M Omer, Mohamed H Ahmed

**Affiliations:** 1 Department of Clinical Pharmacy, Faculty of Pharmacy, Omdurman Islamic University, Khartoum, Sudan; 2 Department of Pharmaceutical Microbiology, Faculty of Pharmacy, Omdurman Islamic University, Khartoum, Sudan; 3 College of Medicine, Ajman University, Ajman, United Arab Emirates; 4 School of Medical Sciences, Örebro University, Sweden; 5 Faculty of Medicine, University of Khartoum, Sudan; 6 Department of Medicine and HIV Metabolic Clinic, Milton Keynes University Hospital NHS Foundation Trust, Eaglestone, Milton Keynes, Buckinghamshire, UK

**Keywords:** assessment, knowledge, attitude, COVID-19, vaccination

## Abstract

**Background:**

Vaccines are an essential part of public health interventions to mitigate the devastating health and non-health impacts of COVID-19 pandemic. Despite the fact that Sudan launched the COVID-19 vaccination program in March 2021, only 10% of the population received their two primary doses of vaccines by the end of May 2022. This delayed uptake of vaccines obviously warrants investigation. Therefore, we have conducted this study to evaluate the knowledge, attitude and acceptance of the general population in Sudan toward COVID-19 vaccines.

**Methodology:**

A descriptive cross-sectional community-based study. The data were collected using an electronic questionnaire from 403 individuals living in Khartoum, Sudan. The data were processed using the Statistical Package for Social Sciences (SPSS), and data analysis was performed using appropriate tests.

**Results:**

51% of the participants were found to have sufficient knowledge about the COVID-19 vaccine, and the knowledge level is higher among those educated beyond the secondary school and those who were employed. Among those unvaccinated, only 47% of the participants expressed their intention to take the vaccine when offered to them. The major reason for not trusting the vaccine is safety concerns expressed by 65.5% of the unvaccinated.

**Conclusion:**

Higher education levels and employment were associated with an increase in sufficient knowledge about the vaccine in around half of the participants. However, most of participants had not taken the vaccine at the time of the study, and the trust in vaccines is not high. Effective interventions by the health authorities are needed to address these issues in order to accelerate the COVID-19 vaccination program in Sudan.

## 1. Introduction

The World Health Organization (WHO) declared COVID-19 as an epidemic and public health emergency of international concern in March 2020 [1]. In the absence of specific antiviral drugs of confirmed effectiveness, vaccination represented the most effective means of prevention. As of early 2021, several vaccines have been approved by the WHO and other health authorities in different parts of the world [2]. Interestingly, the vaccines were developed in record time by the vaccine-manufacturing companies that used innovative technologies [3],[4]. The vaccines developed by Pfizer and Moderna used mRNA technology, while AstraZeneca used adenoviral vector-based vaccines. Vaccines using traditional technologies were also developed and authorized, such as the inactivated vaccine developed by the Chinese company Sinopharm [5] and the subunit vaccine developed by the USA company Novavax [6].

Like almost all countries of the world, Sudan has been affected by the COVID-19 pandemic; the first case was detected in March 2020, and the case fatality rate was estimated to be 6.2% in July 2020 [7]. Sudan was among the first countries in Africa and the Middle East to receive COVID-19 vaccines from international donors. An initial stock of 800,000 doses of Oxford/AstraZeneca vaccines was received in March 2021, through the COVID-19 Vaccines Global Access (COVAX) initiative, to vaccinate health care workers and the elderly people with chronic medical conditions [8]. Subsequent stocks were received by the local health authorities targeting the vaccination of 8 million (20% of the population) by the end of 2021 [9]. Unfortunately, the percentage of the population who received their primary two doses of the vaccine was only around 10% by mid-2022 [10].

For successful implementation of the vaccination programs, it is essential to investigate the knowledge, attitude and acceptance of the public toward the vaccines. Hesitancy toward vaccination is a real challenge for all infectious diseases, and it needs to be seriously addressed by health authorities [11]. Obviously, online misinformation on social media platforms is one of the causes for COVID-19 vaccine hesitancy worldwide [12]. In addition, COVID-19 was shown to be associated with an increase in depression, social media addiction, internet addiction, family conflict and work conflict [13]–[15]. These factors may also contribute in part to whether the individuals will feel confident in taking the vaccine. Therefore, many studies have been conducted to evaluate the knowledge and attitude of the communities toward COVID-19 vaccines in many parts of the world. For example, in Bangladesh the vaccine acceptance level was found to be 61.2% [16]. While, in Ethiopia, the level of knowledge, a positive attitude and the percentage of acceptance among the participants were found to be 74%, 44.7% and 62.6%, respectively [17]. Due to concern about vaccines' possible side effects, the vaccine hesitancy was found to be around 70% in mid-wives in Cyprus [18].

In this study, we aimed to evaluate the knowledge, attitude and acceptance of COVID-19 vaccines among the Sudanese general population. The results of this study will help policy-makers and health authorities in Sudan to carefully evaluate the current and future program of COVID-19 vaccination.

## 2. Materials and methods

### 2.1. Study design and setting

This study is an observational, community-based, descriptive cross-sectional study, which was conducted in Khartoum state during the period from August 2021 to October 2021. Eligible participants included all members of the general population aged 17 to 90 years old who were willing to participate in the study.

### 2.2. Sample size and sampling method

We used the convenience sampling method; the available participants at the time of the study were recruited study participants from the markets, clubs and general streets in Khartoum state, which consists of three areas: Khartoum, Khartoum North and Omdurman.

The sample size was calculated using the formula

$$\begin{array}{c} \begin{array}{c} n=\frac{Z^{2}*p*\left(1-p\right)}{e^{2}} \end{array} \end{array}$$ (1)

where *n* = sample size, *Z* = z score (1.96), *p* = prevalence (50%) and *e* = margin of error (5%);

*n* = (1.96)^2^(0.5)(0.5)/(0.05)^2^, *n* = 384.

To increase the accuracy of the study and reduce the errors that may arise from missed data, we recruited 403 participants in this study.

### 2.3. Data collection method

A structured questionnaire was designed using a Google form to collect data from 403 members of the adult population in Khartoum state. The researchers filled out the electronic questionnaire while they were interviewing the participants. The pilot study was conducted, designated with 10 participants; then, the questionnaire was reevaluated and standardized according to the response of the respondents. All 10 questionnaires of the pilot study were excluded.

The questions of the questionnaire included seven questions about the general sociodemographic information about the participants, nine questions about knowledge about the COVID-19 vaccine and eight questions about acceptance and attitude toward the COVID-19 vaccines.

### 2.4. Scoring method of knowledge questions

The total number of questions in the knowledge section was nine. Each correct answer was given score of 1, and each false answer was given 0; thus, the overall score ranged between 0–9. The median of the score of knowledge was found to be 4 (IQR 1–6); thus, the participants who achieved a score of 4 or more were categorized as having sufficient knowledge, while those who achieved a score of less than 4 were categorized as having insufficient knowledge.

### 2.5. Model and data analysis procedure

The data were analyzed using the Statistical Package for Social Sciences (SPSS software version 26, IBM, Chicago, IL, USA). Descriptive statistics were used to calculate the frequencies and percentages in the form of frequency tables. Chi-square tests and cross-tabulation were used to test the associations between categorical variables (knowledge with sociodemographic characteristics of the participants), with a p-value ≤ 0.05 being considered significant. Furthermore, multivariate binary logistic regression analysis was performed to determine the factors that predict knowledge of the participants toward COVID-19 vaccination. The independent variables introduced in the model were gender, education, academic area and employment, while the dependent variable was knowledge (with binary categories, sufficient and insufficient).

### 2.6. Ethical approval

Consent was obtained from each participant before filling out the questionnaire. Ethical approval was obtained from Omdurman Islamic University, Faculty of Pharmacy (FP–CP–7–2021).

## 3. Results

### 3.1. Sociodemographic characteristic

The distribution of gender among participants was nearly equal, with a slightly higher percentage of males (54%). Most participants were 17–30 years old (60%), single (57%), coming from an urban background (69%) and in possession of a bachelor's degree (51%) in a non-health related academic area (65%). Regarding occupations, students were the most highly represented (31%) of the participants, and retired people were the least (only 3%). Most of participants (82.3%) were healthy with no chronic conditions and non-smokers (78%) (Table 1).

**Table 1.** Sociodemographic characteristics of the participants.

		N	%
Gender	Female	185	45.9
	Male	218	54.1
Age	17–23 Yrs	131	32.5
	24–30 Yrs	110	27.3
	31–37 Yrs	40	9.9
	38–44 Yrs	30	7.4
	≥45 Yrs	92	22.8
Marital status	Divorced	7	1.7
	Married	167	41.4
	Single	229	56.8
Education	Bachelor degree	205	50.9
	Not Educated	26	6.5
	Post-Graduation	42	10.4
	Primary School	35	8.7
	Secondary School	95	23.6
Academic area	Health Related	143	35.5
	Non-Health Related	260	64.5
Employment	Employed	81	20.1
	Retired	10	2.5
	Student	125	31.0
	Unemployed	87	21.6
	Worker	100	24.8
Residence	Rural	54	13.4
	Rural-Urban	70	17.4
	Urban	279	69.2

**Figure 1.**
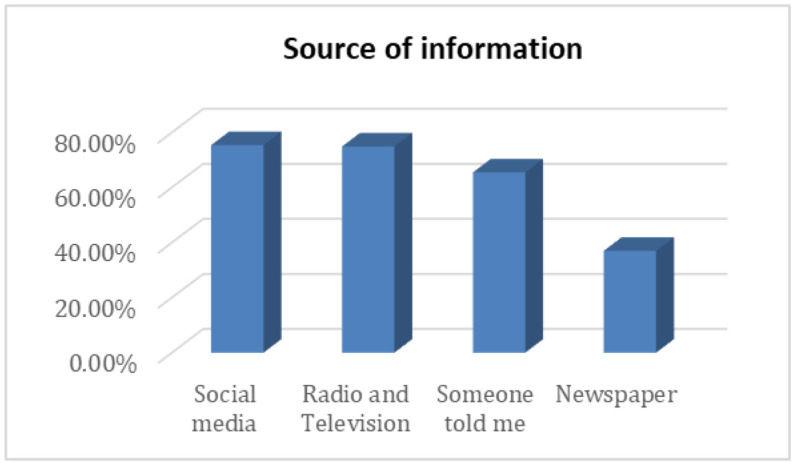
Source of information regarding COVID-19 (n = 403).

Participants used different sources to get information regarding COVID-19 and COVID-19 vaccines. Social media (75%) and radio and television (75%) were the most informative sources of COVID-19-related information to participants (Figure 1).

**Figure 2.**
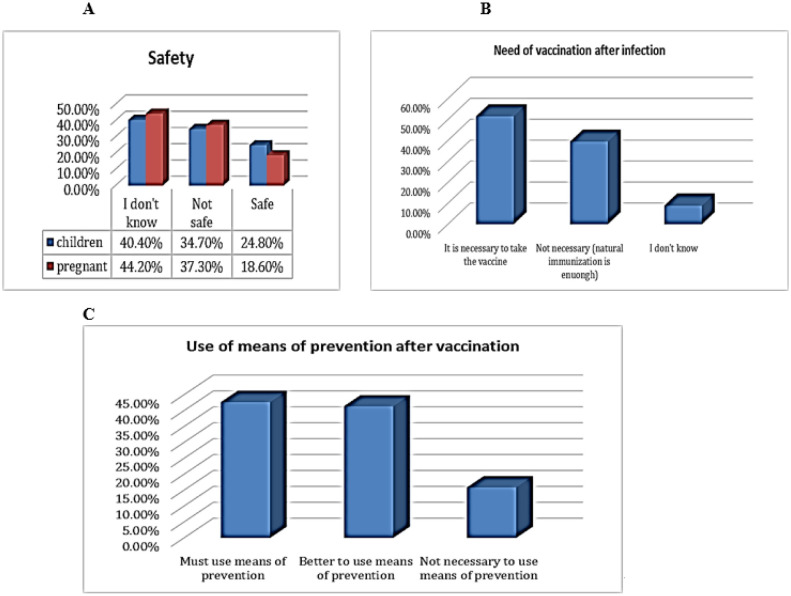
General knowledge of the participants about the vaccine safety, eligibility and the need for using the means of prevention after vaccination; Figure 2A is about the beliefs of participants regarding the safety of the vaccine for children above 5 years and pregnant women (n = 403); Figure 2B is about participants' beliefs regarding the need to take the vaccine after being infected with COVID-19 and the infection has resolved (n = 403); Figure 2C shows the participants' beliefs regarding the need to use means of prevention after being vaccinated (n = 403).

Most participants considered that the vaccine is available and free (58%) and that the vaccine is given in two primary doses (69%). However, about 40.4% and 44.2% of participants did not know whether the COVID-19 vaccine is safe for pregnant women and children, respectively (Figure 2A). About 52% participants believed that it is necessary to take the vaccine after recovery from a previous infection (Figure 2B). About 84% agreed that it is important to use other means of prevention after being vaccinated, and 16% of them reported that it is not necessary to use the means of prevention (Figure 2C).

As shown in Table 2, the most known vaccine-related side effect according to participants was headaches (71.5%), followed by inflammation (50.9%). More than half of the participants (53.1%) considered that there is no benefit in taking the vaccine while the person has symptoms of the disease (Table 2); 51.1% had sufficient knowledge about the COVID-19 vaccine. The median score of knowledge of participants about the safety of the COVID-19 vaccine was found to be 4 (with a 95% confidence interval between 1 and 6).

**Table 2.** Participants' knowledge regarding safety of the COVID-19 vaccine.

	Yes		No		I don't know	
	N	%	N	%	N	%
The vaccine causes allergy	166	41.2	64	15.9	173	42.9
The vaccine causes local inflammation at the site of injection	205	50.9	41	10.2	157	39
The vaccine causes diarrhea or vomiting	138	34.2	79	19.6	186	46.2
The vaccine causes headaches	288	71.5	18	4.5	97	24.1
The vaccine causes breathlessness	142	35.2	77	19.1	184	45.7
The vaccine causes persistent bleeding under the skin	35	8.7	127	31.5	241	59.8
The vaccine causes death	118	29.3	130	32.3	155	38.5
Benefit of vaccination while the person is sick with the disease	105	26.1	214	53.1	84	20.8

**Table 3.** Cross-tabulation between participants' knowledge about vaccines and their sociodemographic data.

Variable		Insufficient Knowledge n = 197	Sufficient Knowledge n = 206	P-value
Gender	Male	114	104	0.083
	Female	83	102	
Education degree	Not Educated	19	7	0.000*
	Primary School	20	15	
	Secondary School	64	31	
	Bachelor's	79	126	
	Post-Graduation	15	27	
Academic area	Health Related	148	112	0.000*
	Non-Health Related	49	94	
Employment	Employed	30	51	0.002*
	Unemployed	44	43	
	Worker	65	35	
	Student	53	72	
	Retired	5	5	

*Note: P value of <0.05 considered statistically significant.

**Figure 3.**
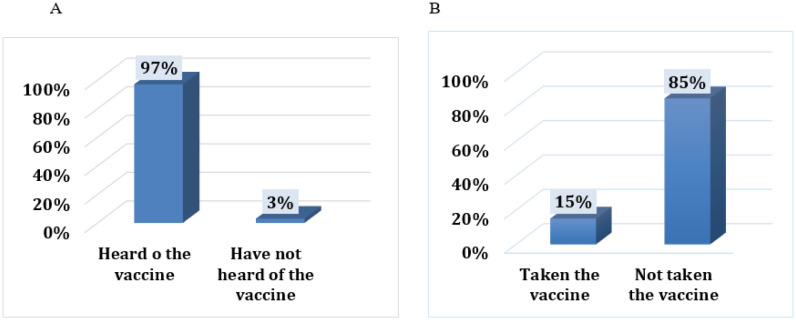
Prevalence of vaccination among the participants and their attitudes toward COVID-19 vaccines: A shows the percentage of participants who had heard of the vaccine (n = 403). B is about the percentage of COVID-19 vaccination among participants (n = 403).

Almost all participants (97%) had already heard of the COVID-19 vaccines (Figure 3A). Most participants (85%) had not taken the vaccine at the time of the research (Figure 3B). The decision of 55% of participants to take the vaccine had not been affected by circulated rumors about the vaccine side effects. Furthermore, 47% of participants who did not take the vaccine were willing to take the vaccine when offered to them.

About 39% of the participants who took the vaccines were encouraged by their friends, and 36% by their families. About 36% of the total participants reported that they would prefer the AstraZeneca vaccine, while 23% preferred Pfizer's vaccine if they were given the choice; 54% of the participants had trust in the effectiveness of the vaccines. The most common reasons for not trusting the COVID-19 vaccine among participants were found to be concerns about the safety of the vaccines (65.5%), insufficient information about the vaccine (67.6%) and the risk of getting the disease through the vaccine (42%).

### 3.2. Chi-square and logistic regression analysis

Using chi-square testing to determine the association between knowledge and other variables, we found that the score of knowledge is significantly associated with education degree (sufficient knowledge was significantly associated with those who had a bachelor degree, P-value: 0.000), academic area (sufficient knowledge was significantly associated with being in the medical field, P-value: 0.000) and employment (sufficient knowledge was significantly associated with being employed, P-value: 0.002) (Table 3).

Logistic regression revealed that females are 1.5 times more likely to have sufficient knowledge than males (OR = 1.9 (CI: 0.9–2.4); p = 0.073), while those with a degree were 2.7 times more likely to have sufficient knowledge than those who completed their education up to the secondary school level (OR = 2.7 (CI: 1.5–4.7); p = 0.001). Furthermore, education was the only predictor for the participant's knowledge regarding the safety of COVID-19 vaccines (Table 4).

**Table 4.** Predicting participant's knowledge using binary logistic regression testing.

	B	P value	Odds ratio	CI of (OR)
Gender	0.424	0.073	1.5	0.9–2.4
Education	0.997	0.001	2.7	1.5–4.7
Academic Area	0.456	0.079	1.5	0.9–2.6
Employment	0.588	0.089	1.8	0.9–3.5

## 4. Discussion

In this study, we found that the knowledge of the population about the danger of the COVID-19 disease was high; about 83% of the participants believed that the disease is real and dangerous, and 92.8% of them thought that the disease is caused by viral infection. This is higher than what was reported by other studies conducted during the first year of the pandemic; for example, in Qatar, only 52.31% of participants believed that the disease is real [19]. We also found that 51.1% of the participants had sufficient knowledge about the COVID-19 vaccine, while, in Ethiopia, only 33.9% of participants had a sufficient score of knowledge, which could be attributed to the fact that the Ethiopian study was applied in rural areas with low electrical supply and internet connection; as a result, they had limited access to COVID-19 information [20]. 75% of the participants reported that they gained their information from the radio and television and 37% of them from newspapers. Importantly, in undergraduate pharmacy students in Zambia, 48.77% gained information from the internet, while 39.75% gained it from television [21]. This can be attributed to the fact that the internet delivers information quicker and cheaper, particularly for young adults, in comparison with newspapers [19].

Vaccine acceptance is crucial in any successful campaign of immunization. Despite the fact that 97% of the participants in our study had already heard of the vaccine, only 15% of them had received the vaccine. Only 47% had the intention to take the vaccine if it became available, and only 54% were found to trust the vaccine. According to studies conducted in 2021 in some Arab countries, the acceptance of COVID-19 vaccines was generally low and estimated to be 29.4% of the population in Kuwait and 20% in Qatar [19],[22]. In Jordan, it was found that there is a low percentage of acceptance that varies between 36.8–37.4% [23],[24]. Furthermore, in Saudi Arabia, 64.7% accepted to take the COVID-19 vaccine, 7.0% refused to take the COVID-19 vaccine and 28.2% were unsure about taking the COVID-19 vaccine if it became available [25]. In another study conducted in 42 different countries, including African and Middle Eastern countries, approximately one-third of the participants were reluctant to receive the COVID-19 vaccine, and the majority (67%) intended to receive the COVID-19 vaccine [26]. Importantly, data showed high rates of acceptance of the vaccines in Asian countries. For instance, the acceptance rate of vaccines in Malaysia was 83%, 90% in China and 55% in Russia [27].

It is important that people have trust and confidence in taking the vaccine. In this study, 46% expressed that they do not trust the vaccines, and 67.6% were worried because there was not enough information regarding the vaccine, while 65.5% were concerned about the safety of the vaccine. Similarly, 45% of the participants had refused to take the vaccine due to worries about side effects and a belief that the vaccine can lead to infertility and death. This is not surprising because, in 42 different countries, it was found that the most common reasons given by participants for avoiding COVID-19 vaccination was mistrust and uncertainty about the vaccine, and another reason was concern about the adverse effects of the vaccine [26]. In Japan, it was reported that 58% requested additional information about the vaccine [23]. About 78.5% of the pharmacy students in Zambia were concerned about the possible adverse effects of COVID-19 vaccines, and 71.1% thought that the COVID-19 vaccines did not pass through all stages of the clinical trials; hence, they were concerned with safety issues [21].

In our study, the score of knowledge for females (68.6%) was found to be higher than males' (57.3%). A higher vaccine hesitancy was associated with the female gender in Qatar [19]. In addition, in the current study, the percentage of the score of knowledge for divorced persons (85.7%) was found to be the highest, followed by single participants (66.8%); the percentage of the score of knowledge for participants who were employed was found to be 70.4%, while those for those who were retired and students were 70.0% and 69.6%, respectively. Similarly, in a study conducted in Zambia, those who were married and those who were unemployed were less likely to accept the COVID-19 vaccine, and it was concluded that the higher acceptance rate among employed persons may have been due to a recommendation of vaccination by their employers [21]. In this study, the percentage of the score of knowledge for those who had post-graduate degrees was 76.2%, followed by bachelor's holders at 70.7%. Furthermore, obtaining a degree was the only predictor for knowledge, as this was shown to be associated with 2.7 times more sufficient knowledge about the vaccine than those who completed their education up to the secondary school level (OR = 2.7 (CI: 1.5–4.7; p = 0.001). This clearly reflects the importance of education among the community members.

The percentage of the score of knowledge for participants studying or working in a health-related sector was found to be 79%, compared to 53.5% in other sectors. This could be attributed to the need of participants belonging to the health sector to often visit medical facilities, such as hospitals and primary care services. Therefore, they were aware that their susceptibility to infection is higher than others; another possible reason is that highly educated participants and those related to the health sector have a higher likelihood to get information from trusted sources. This was also shown in China, where 79.1% of students studying health-related courses were found to be willing to take the COVID-19 vaccine [28]. This can be attributed in part to the fact that COVID-19 vaccine acceptance was higher in countries with a very high to medium Human Development Index [29].

Factors that are significantly associated with knowledge in this study included gender, marital status, employment, academic area and educational degree. This is comparable to what was detected in other studies that were conducted in Ethiopia and Zambia, in which the same factors were found to be significantly associated with knowledge about the COVID-19 vaccine [20],[21]. It is worth mentioning that different studies about COVID-19 vaccination in Sudan showed different outcomes. For instance, Elbadawi et al. showed that over three-fifths of healthcare workers agreed that COVID-19 vaccination was important and should be mandatory, while 62.8% had concerns regarding side effects of the vaccine and 57.3% believed insufficient trials were conducted [30]. Yassin et al. also showed a high rate of acceptance of vaccination among healthcare workers in Sudan of 63.8%, while only 22.7% got vaccinated [31]. Vaccine hesitancy among Sudanese medical students was found to be 44.2% [32]. Only 31% of individuals with diabetes in Eastern Sudan received the vaccine [33]. Vaccine hesitancy can be attributed to the worries about long-term complications [34]. Therefore, many lessons can be learned by the Ministry of Health in Sudan regarding the preparedness to cope with COVID-19 and a better policy to decrease vaccine hesitancy. For example, nations have a natural level of maximum vaccinable people (70% of population), so the government should aim to increase expenditure in health systems and have better public governance; and, prevention measures should start in summertime and there should be the adoption of new technology [35]–[39].

This study is not without limitations. First, the cross-sectional study design may not allow for the determination of the relationship between the outcomes and other factors. The study was only conducted in Khartoum state, so the findings cannot be generalized to the whole population of Sudan. Second, the study was carried out using the convenient sampling technique; therefore, the subjective selection bias was not avoidable, which can affect the study's internal validity.

## 5. Conclusions

Half of the participants revealed sufficient knowledge regarding COVID-19 vaccines. Most participants had not taken the vaccine at the time of the study. Knowledge had statistically significant association with educational level and employment. The major determinant of the knowledge is the educational level, and the major reason for not trusting the vaccine is concern about the safety of the vaccine. The results of this study would contribute to the information needed by the health authorities to adopt effective strategies, effective interventions and promotion campaigns to accelerate the COVID-19 vaccination program in Sudan. The role of family and primary care physicians is essential in improving the vaccination campaign, and also in increasing health education about the benefits of vaccination.
